# *Agrobacterium tumefaciens*-Mediated Transformation of NHEJ Mutant *Aspergillus nidulans* Conidia: An Efficient Tool for Targeted Gene Recombination Using Selectable Nutritional Markers

**DOI:** 10.3390/jof7110961

**Published:** 2021-11-12

**Authors:** Virginia Casado-del Castillo, Andrew P. MacCabe, Margarita Orejas

**Affiliations:** Instituto de Agroquímica y Tecnología de Alimentos (IATA), Consejo Superior de Investigaciones Científicas (CSIC), c/Catedrático Agustín Escardino Benlloch 7, 46980 Paterna, Valencia, Spain; virginiacasado@usal.es (V.C.-d.C.); morejas@iata.csic.es (M.O.)

**Keywords:** *Aspergillus nidulans*, conidia, fungal transformation, ATMT, pCAMBIA-derived vectors, targeted gene deletion/disruption, nutritional (*pyrG*/*pyr4*) and colour (*wA*) selectable markers, mutagenesis, NHEJ, Δ*nkuA* mutants

## Abstract

Protoplast transformation for the introduction of recombinant DNA into *Aspergillus nidulans* is technically demanding and dependant on the availability and batch variability of commercial enzyme preparations. Given the success of *Agrobacterium tumefaciens*-mediated transformation (ATMT) in diverse pathogenic fungi, we have adapted this method to facilitate transformation of *A. nidulans*. Using suitably engineered binary vectors, gene-targeted ATMT of *A. nidulans* non-homologous end-joining (NHEJ) mutant conidia has been carried out for the first time by complementation of a nutritional requirement (uridine/uracil auxotrophy). Site-specific integration in the Δ*nkuA* host genome occurred at high efficiency. Unlike other transformation techniques, however, cross-feeding of certain nutritional requirements from the bacterium to the fungus was found to occur, thus limiting the choice of auxotrophies available for ATMT. In complementation tests and also for comparative purposes, integration of recombinant cassettes at a specific locus could provide a means to reduce the influence of position effects (chromatin structure) on transgene expression. In this regard, targeted disruption of the *wA* locus permitted visual identification of transformants carrying site-specific integration events by conidial colour (white), even when auxotrophy selection was compromised due to cross-feeding. The protocol described offers an attractive alternative to the protoplast procedure for obtaining locus-targeted *A. nidulans* transformants.

## 1. Introduction

Genetic transformation is an essential tool for identifying as well as manipulating gene function. Over the 40 years since the first clear demonstration of transformation of a filamentous fungus (*Neurospora crassa*; [1]), several methods for introducing genetic material into fungal cells have been developed (reviewed in [2]). Despite this, the technique employed by Case et al. [1], namely the generation and transformation of protoplasts, is still widely used especially in the model filamentous fungi-including *Aspergillus nidulans* [3]. Empirical modifications of the technique have, in general, either sought to improve protoplast generation, transformation efficiencies, the frequency of homologous transformation, or been directed towards the transformation of specific fungal species, but the methodology itself remains founded on the enzymatic deconstruction of the fungal cell wall and consequent liberation of osmotically fragile protoplasts, the appropriate treatment of which can result in the uptake and expression of exogenous DNA. Cell wall deconstruction is achieved by incubation of fungal biomass with commercial mixtures of cell wall degrading enzymatic activities. This treatment is, however, difficult to control, and its outcome can be unpredictable since different enzyme mixtures work to different degrees on different fungal species and their various developmental stages. Enzyme cocktail availability and/or batch variability constitute additional problems, and of particular note in this regard was the cessation of production of Novozyme 234 [4] and Vinoflow FCE [5] requiring subsequent trials with other enzyme mixtures as substitutes.

*Agrobacterium tumefaciens*-mediated transformation (ATMT) of filamentous fungi is an alternative technique originally developed with a view to improving transformation efficiency. It has been found to work on various fungal structures—conidia, hyphae, protoplasts, fruiting bodies—and also has the advantage of almost exclusively introducing only the exogenous DNA sequences of interest along with a selectable marker, thus avoiding the concomitant incorporation of unnecessary vector DNA into the fungal genome [2,6,7]. ATMT has been used across a wide range of fungal species distributed in several phyla (recently reviewed [8]), successful transformation being manifested in many cases (but not uniquely) by the acquisition of resistance to an antibiotic (e.g., hygromycin B, conferred by the *hph* gene [9]). As pointed out by Idnurm et al. [8], ATMT has found wide application in insertional mutagenesis and reverse genetics in pathogenic fungi, but it has barely been used in the model filamentous fungi *N. crassa* and *A. nidulans*, an exception in the latter case being an assessment of gene silencing by d-siRNA [10].

The current study is directed to exploiting three properties/resources of *A. nidulans* to develop a reliable ATMT alternative to protoplast transformation that would facilitate the use of reverse genetics techniques in this fungus for the analysis of gene or promoter function, or the modulation of gene expression (gene deletion/disruption, allele replacement, promoter swapping, protein tagging, (over)expression of recombinant gene cassettes): whereas *A. nidulans* protoplasts tend to be multinucleate and therefore initially result in the generation of heterokaryotic transformants, *A. nidulans* conidia are uninuclear and hence have the potential to yield homokaryotic transformants directly; since site-specific genomic targeting is an important attribute for performing reverse genetics, it would be of interest in this context to examine the use of NHEJ strains as ATMT hosts; several functional orthologous gene expression cassettes are available that complement corresponding auxotrophies in *A. nidulans*, thus making nutrient marker selection a possibility in ATMT of this fungus. By incorporating these features in ATMT of *A. nidulans* we have (i) efficiently obtained specific gene deletion mutants and (ii) visually identified site-specific integration transformants independently of auxotrophic selection.

## 2. Materials and Methods

### 2.1. Strains, Media, Growth Conditions, and Reagents

*A. tumefaciens* strain AGL1 (AGL0, *recA*::*bla*, pTiBo542 (Δ)T, Mop^+^) [11], resistant to both carbenicillin (Cb^R^) and rifampicin (Rif^R^), was used to transform *A. nidulans* conidia. *Escherichia coli* DH5α was used for cloning experiments and plasmid amplification [12]. The *A. nidulans* strains used are detailed in Table 1.

*E. coli* and *A. tumefaciens* were grown in Luria–Bertani (LB) medium. Details of the specific growth conditions and antibiotics used for each are given in the corresponding text. *A. nidulans* conidia for ATMT were harvested in sterile distilled water (by gentle surface scraping) from complete medium (CM) plates [15,16], containing supplements where specifically required, after incubation at 37 °C for 7 days. Conidia were stored at 4 °C, and titres were determined using a Neubauer counting chamber. Prior to use, conidia were pelleted by centrifugation for 10 min at 1000× *g* at room temperature and resuspended in *A. tumefaciens* induction medium (*At*-IM).

All solidified media contained 1.5% agar. Solutions and media used for ATMT of *A. nidulans* conidia are detailed in Supplementary Table S1.

The following solutions were prepared and stored in darkness as aliquots at −20 °C: acetosyringone stock at 200 mM (1000×) in absolute ethanol—vortex well upon thawing to ensure dissolution; carbenicillin stock at 100 mg/mL (1000×) in sterile distilled water; cefotaxime stock at 200 mM (1000×) in sterile distilled water; kanamycin stock at 25 mg/mL (500×) in sterile distilled water; rifampicin stock at 10 mg/mL (400×) in methanol.

Sterile cellulose nitrate filters (0.45 µm pore size, filter diameter 4.7 cm) were supplied by Sartorius Stedim Biotech GmbH (Goettingen, Germany).

### 2.2. A. tumefaciens-Mediated Transformation

#### 2.2.1. Preparation of *A. tumefaciens* Competent Cells

In the following procedure all incubations took place in darkness, and manipulations were undertaken avoiding exposure of the cells to light. Cells were streaked from a −80 °C glycerol stock of *A. tumefaciens* AGL1 onto LB agar medium supplemented with 100 µg/mL of carbenicillin and incubated at 28 °C for 2–3 days. An isolated colony was picked and used to inoculate 100 mL of LB supplemented with rifampicin (25 µg/mL) and carbenicillin (100 µg/mL), and the culture was grown at 28 °C and 200 rpm for 16 h. The culture was pre-chilled on ice for 10 min with gentle swirling, and all subsequent steps were carried out at 4 °C. Cells were harvested by centrifugation at ~3500× *g* for 5 min at 4 °C, and the supernatant was removed. The cell pellet was resuspended in 50 mL ice-cold 10% (*v*/*v*) glycerol and recovered by centrifugation under the same conditions. This process was repeated twice more, and cells were finally resuspended in 1 mL of ice-cold 10% glycerol (*v*/*v*) from which 40 µL aliquots were made and stored at −80 °C until needed.

#### 2.2.2. Transformation of *A. tumefaciens* by Electroporation

The following procedure was undertaken with minimal exposure to light. An aliquot of competent AGL1 cells was thawed on ice and transformed with 50–200 ng (in a volume of <4 µL) of the appropriate Ti binary plasmid by electroporation in an ice-chilled cuvette using a Bio-Rad MicroPulser set to the ‘Agr’ program (2.2 kV). Recovery of the cells was undertaken in 1 mL of LB incubated for 4 h at 28 °C with orbital shaking at 300 rpm. Selection for transformants was done by spreading aliquots of the recovery mix on LB agar plates containing the appropriate antibiotic (50 µg/mL kanamycin in this work) and incubating at 28 °C for 48 h in darkness.

#### 2.2.3. Transformation of *A. nidulans* Conidia by *A. tumefaciens*

The following procedure was undertaken with minimal exposure to light. A colony of the transformed *A. tumefaciens* cells containing the desired binary plasmid was picked and inoculated into 2 mL of *At*-minimal medium (*At*-MM) containing kanamycin (50 µg/mL), and this was incubated in darkness at 28 °C and 250 rpm for 48 h. The OD_600_ of the culture was measured, and cells were recovered under sterile conditions by centrifugation at 2400× *g* for 10 min at room temperature. The pellet was resuspended in *At*-IM supplemented with 50 µg/mL kanamycin and 200 µM acetosyringone to yield a final OD_600_ of 0.15. A total of 2 mL of this culture was incubated in darkness at 28 °C and 250 rpm for 6 h, after which the OD_600_ was readjusted to 0.15 with *At*-IM containing kanamycin and acetosyringone. Equal volumes (e.g., 1 mL) of this culture and a suspension of *A. nidulans* conidia in *At*-IM at a known titre (see above and Section 3.1 for details) were mixed yielding a final co-culture volume of 2 mL. Aliquots (200 µL) were spread on a total of ten cellulose nitrate filters that had been placed 3 h previously on Petri dishes containing solidified *At*-IM supplemented with kanamycin and acetosyringone. These plates were then incubated in darkness at 24 °C for 48 h. After this period, each filter was carefully lifted from its plate and cut into four segments under sterile conditions. The segments were placed three per plate on appropriately supplemented (where necessary) *A. nidulans* solidified minimal medium (MM, [16]) containing cefatoxime (200 µM) to kill the *A. tumefaciens* cells, and these were incubated at 37 °C. Plates were inspected daily for the growth of transformants.

### 2.3. Nucleic Acid Manipulations

#### 2.3.1. General

Genomic DNA was extracted from 0.2–0.5 g of *A. nidulans* mycelium that had been finely ground under liquid nitrogen and then resuspended in 600 µL of 50 mM Tris pH 7.5, 20 mM EDTA (pH 8), to which was added 40 µL of SDS (10% *w*/*v*). The mixture was incubated at 65 °C for 30 min, after which 180 µL of lysis solution (preparation: 6 mL of 5 M potassium acetate + 1.15 mL glacial acetic acid + 2.85 mL distilled water; i.e., 3 M K^+^, 5 M acetate) was added, and the whole was maintained on ice for 30–60 min. Centrifugation was performed at 16,000× *g* at 4 °C for 15 min, and 650 µL of supernatant was recovered and transferred to a 2 mL Eppendorf tube. Two volumes of cold ethanol (−20 °C) were added and mixed gently, and the whole was maintained at −20 °C for at least 1 h. Centrifugation was repeated as before, the supernatant was eliminated, and the pellet was carefully washed with 70% ethanol at room temperature. After air drying to eliminate ethanol, the pellet was dissolved in 100 µL TE (10 mM Tris, 1 mM EDTA pH 7.5), to which 3 µL of 10 mg/mL RNAaseA (DNAase free) was added, and the whole was incubated at 37 °C for 30 min. After this period, 10 µL of 3M sodium acetate pH 5.2 was added, mixed well, and two volumes of cold ethanol were layered on top. gDNA was spooled out with a pipette tip. The recovered DNA was washed at room temperature with 70% ethanol as before and air dried. The pellet was redissolved in 50–100 µL TE. (This protocol is based on a method used in the Scazzocchio lab).

Unless otherwise stated, PCR reactions were performed using blunt-end yielding Phusion High-Fidelity DNA polymerase (Thermo Fisher Scientific, Waltham, MA, USA) according to the manufacturer’s instructions. Restriction enzymes were supplied by Thermo Fisher Scientific, Roche (Basel, Switzerland), and New England Biolabs Inc. (Ipswich, MA USA). Oligonucleotide primers (Metabion International AG, Planegg, Germany) used in this study are detailed in Supplementary Table S2.

#### 2.3.2. Construction of ATMT Binary Vectors

**Figure 1:**
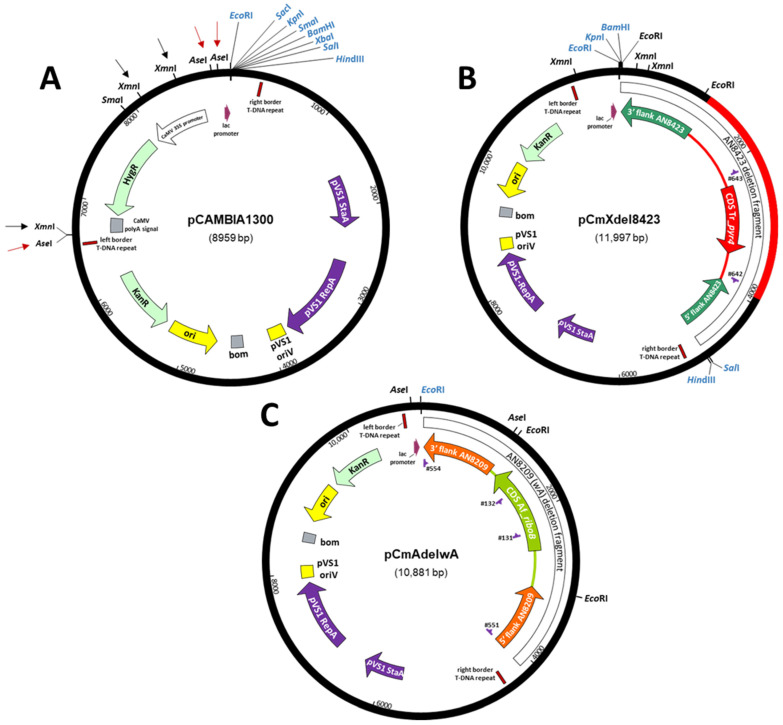
Plasmids used in this work. (**A**) Construction of binary vectors for implementation of ATMT in *A. nidulans.* *A. tumefaciens* binary vector pCAMBIA1300 carrying the hygromycin and kanamycin resistance genes. Black and red arrows show the restriction sites for *Xmn*I and *Ase*I: digestion with *Xmn*I followed by recircularisation yielded pCAMBIA-mini^XmnI^; digestion with *Ase*I followed by recircularisation yielded pCAMBIA-mini^AseI^. Restriction sites in the MCS are shown in blue. (**B**) Modified binary vector for the deletion of AN8423 by ATMT. pCmXdel8423 comprises the *A. tumefaciens* binary vector pCAMBIA-mini^XmnI^ into which has been cloned the AN8423 deletion fragment containing the Tr_*pyr4* expression cassette that complements the *pyrG89* mutation in *A. nidulans*. Restriction sites of the binary vector MCS are shown in blue. (**C**) Modified binary vector for integration of Af_*riboB* in *wA* by ATMT. pCmAdelwA comprises the *A. tumefaciens* binary vector pCAMBIA-mini^AseI^ into which has been cloned the *wA* (AN8209) deletion fragment containing the Af_*riboB* expression cassette that complements the *riboB2* mutation in *A. nidulans.* The *Eco*RI site of the binary vector MCS is shown in blue.

For *A. nidulans* ATMT, modified pCAMBIA1300 plasmids were generated in which DNA fragments corresponding to the expression cassette containing the bacterial hygromycin B resistance gene (*hph*) were eliminated whilst retaining the T-DNA borders and the *lac*Zα fragment (X-gal screening) (vide infra Figure 1A). Two restriction digests of pCAMBIA1300 (https://cambia.org/welcome-to-cambialabs/cambialabs-projects/cambialabs-projects-legacy-pcambia-vectors-pcambia-legacy-vectors-1/ (accessed on 9 November 2021)) were carried out independently using *Xmn*I (removes 1.78 kb) on the one hand and *Ase*I (removes 2.18 kb) on the other. In each case the vector fragments (7.17 kb and 6.77 kb) were purified by agarose gel electrophoresis (Monarch gel extraction kit, New England BioLabs Inc.) and re-circularised to yield plasmids pCAMBIA-mini^XmnI^ and pCAMBIA-mini^AseI^, respectively.

#### 2.3.3. PCR of Conidia

PCR analysis of *A. nidulans* transformants directly from their conidia was done as follows. DNA was released from conidia using the microLYSIS-PLUS kit (Microzone, Brighton, UK) following the manufacturer’s instructions. Upon completion of the protocol, an equal volume (20 µL) of 0.1× TE was added to each extract, and the whole was centrifuged at 12,000× *g* for 5 min at room temperature. A total of 10 µL of supernatant was then added to a PCR reaction (final volume 32.5 µL) performed with Top-Taq polymerase (Bioron, Ludwigshafen, Germany) following the latter’s recommendations using oligonucleotide primer pairs 641/642 and 643/644, an annealing temperature of 58.5 °C, and an extension time of 90 s. Products were analysed on 0.8% agarose gels using RedSafe (iNtRON Biotechnology, Burlington, MA, USA) nucleic acid staining solution.

#### 2.3.4. Deletion of AN8423

DNA fragments upstream and downstream of locus AN8423 were obtained by amplification (annealing temperature of 67 °C and extension time of 45 s) off *A. nidulans* AR271 gDNA using oligonucleotide primer pairs 573/574 and 575/576, and these were subsequently cut with *Kpn*I/*Sal*I and *Sal*I/*Spe*I, respectively. Agarose gel-purified fragments (Illustra GFX PCR DNA and Gel Band Purification Kit, GE Healthcare, Chicago, IL, USA) were ligated (T4 DNA ligase 5 U/µL) with 100 ng of *Kpn*I/*Spe*I cut pBluescript SK(+) in a 1:1:1 molar ratio in a reaction volume of 30 µL at 16 °C overnight, yielding plasmid pE520. The Tr_*pyr4* expression cassette was released from pFG1 [17] as a ~2.7 kb *Sal*I fragment and cloned between the AN8423 upstream and downstream sequences in the unique *Sal*I site of pE520 to yield pE527. The complete deletion cassette can be released from pE527 as a 4.81 kb *Spe*I fragment by virtue of recognition sites for this enzyme present in oligonucleotide primers 573 and 576.

#### 2.3.5. Integration at *wA*

In order to construct a recombinant cassette to target integrations to the *wA* locus (AN8209), DNA fragments upstream and downstream of the structural gene were obtained by amplification off *A. nidulans* gDNA (strain AR271) using oligonucleotide primer pairs 551/552 and 553/554, and these were subsequently cut with *Bam*HI/*Eco*RI and *Eco*RI/*Sal*I, respectively. The purified fragments were ligated with 100 ng of *Bam*HI/*Sal*I digested pBluescript SK(+) in a 1:1:1 molar ratio yielding plasmid pWHITE, into which the *A. fumigatus riboB* expression cassette (Af_*riboB*; released from pTN2 [13] as a 2 kb *Eco*RI fragment) was subsequently inserted, producing pWHITE-*riboB.* The design of primer 552 situates unique *Eco*RI and *Eco*RV restriction sites between the AN8209 flanking sequences to facilitate the introduction of those DNA sequences destined for integration in the *wA* locus. For use in ATMT, the 5’AN8209-Af_*riboB*-3′AN8209 insertion cassette was amplified off pWHITE-*riboB* by high-fidelity PCR using primers 551 and 554 and inserted by blunt-end ligation into the binary vector pCAMBIA-mini^AseI^ (see above) cut with *Sma*I, resulting in plasmid pCmAdelwA.

## 3. Results

### 3.1. Adaptation of ATMT to A. nidulans and Deletion of AN8423 as a Prototype of a Targeted Gene

The generation and phenotypic analysis of null mutants is an important aid to gene characterization and is often achieved by targeted replacement of a DNA sequence of interest by a selectable marker. Successful ATMT of a fungus requires the construction of a binary vector to achieve the transfer and expression of the transforming DNA in the recipient cell along with an appropriate marker to enable the selection of transformants. Whilst hygromycin resistance is a frequently used selection system in binary vectors, its usefulness in *A. nidulans*, in particular, is limited due to the variable but relatively high intrinsic resistance to this antibiotic reported in diverse strains of this species [18,19,20]. By contrast, selection in *A. nidulans* based on nutritional markers such as the activity of orotidine-5′-phosphate decarboxylase (encoded by *pyrG*) has a long history of reliable use, and a *Trichoderma reesei pyr4* expression cassette (Tr_*pyr4*; [17]) is able to complement the *pyrG89* loss-of-function mutation and confer uridine/uracil prototrophy. Site-specific genomic targeting has also been greatly enhanced by the development of non-homologous end-joining (NHEJ) mutants, and a number of such *A. nidulans* strains have been developed that also carry auxotrophic mutations including *pyrG89* [13].

We have utilised the features mentioned above to develop ATMT for gene deletion in *A. nidulans* using AN8423 as a test locus (putatively encodes a transmembrane transport protein). A gene deletion construct was designed comprising the functional Tr_*pyr4* element (2691 bp) flanked by targeting sequences corresponding to the upstream (945 bp) and downstream (1178 bp) regions of the AN8423 CDS (i.e., 5′AN8423-Tr_*pyr4*-3′AN8423), and this was cloned into pBluescript SK(+) to yield plasmid pE527 (see Section 2.3.4 for details). Given the unsuitable nature of hygromycin selection in *A. nidulans*, the binary vector pCAMBIA1300 (https://cambia.org/welcome-to-cambialabs/cambialabs-projects/cambialabs-projects-legacy-pcambia-vectors-pcambia-legacy-vectors-1/ (accessed on 9 November 2021); Figure 1A) was modified by *Xmn*I digestion (total) and re-circularization to eliminate 1.78 kb of sequence encompassing the *E. coli* hygromycin B resistance (*hph*) expression cassette to obtain plasmid pCAMBIA-mini^XmnI^. The 5′AN8423-Tr_*pyr4*-3′AN8423 deletion construct (recovered as an *Spe*I fragment from pE527) was subsequently inserted at the *Xba*I site of the MCS of this plasmid, yielding pCmXdel8423 (Figure 1B) that was used to transform *A. tumefaciens* strain AGL1 by electroporation.

**Figure 2:**
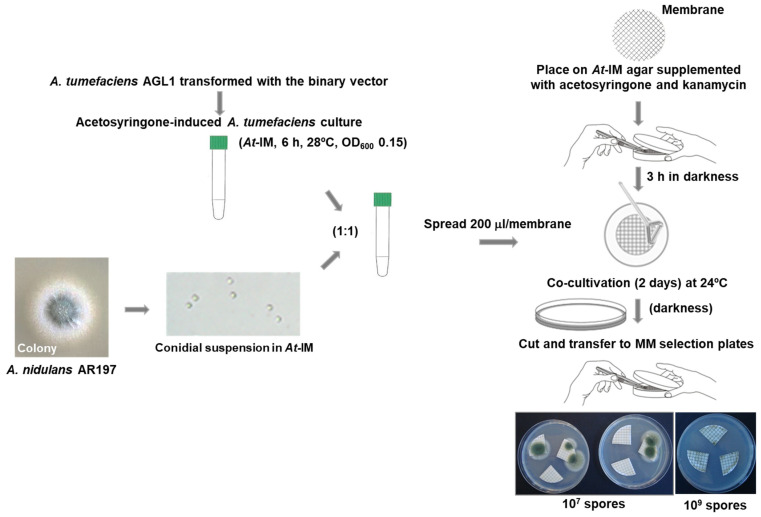
Schematic overview of ATMT of *A. nidulans* conidia. Transformants were obtained after 3 days’ growth at 37 °C on *Aspergillus* selective MM plates supplemented with cefotaxime. *At*-IM: induction medium supplemented with kanamycin and acetosyringone.

In addition to obtaining an appropriate binary vector, certain technical parameters of ATMT were fine-tuned to optimise the technique to the particular fungal cells to be transformed. Given the convenience and benefits of *A. nidulans* conidia as a potentially transformable tissue, a series of co-cultures of *A. tumefaciens* cells and conidia were carried out on cellulose nitrate filters placed on *At*-IM plates mixing equal volumes (100 µL) of acetosyringone-induced pCmXdel8423-transformed *A. tumefaciens* cells (OD_600_ = 0.15) and conidia of the *A. nidulans* NHEJ uridine auxotroph AR197 (Δ*nkuA::argB*, *argB2*, *pyrG89*, *pyroA4*), varying both the titre of the conidial suspension used as well as the duration of the co-culture incubation (48 or 72 h). After co-culture, filters were cut into quadrants that were transferred to pyridoxine-supplemented MM plates (three quadrants per plate) and incubated at 37 °C to promote the growth of uridine prototrophic transformants. A co-culture period of 48 h yielded no transformants when conidial titres (conidia/mL) of 10^6^ and 10^9^ were used. By contrast, use of a conidial suspension of titre 10^8^ yielded 31 transformants, which was three-fold greater than that obtained using a titre of 10^7^. Extension of co-culture to 72 h only resulted in greater bacterial proliferation and failed to yield more transformants. In addition, no notable differences were seen in the number of transformants obtained when either fresh conidia or those stored in suspension at 4 °C for 2 months were used. Given these observations, our future ATMTs were carried out by spreading 200 µL aliquots of co-culture mixture comprising 100 µL of bacterial cells (OD_600_ 0.15) and 100 µL of *A. nidulans* conidia at a titre of 10^8^/mL (i.e., a 1:1 ratio of these components) on individual cellulose nitrate filters and co-cultivating on *At*-IM for 48 h at 24 °C in darkness prior to the transfer of filter quadrants to fungal selection plates. A schematic summary of the procedure is presented in Figure 2.

**Figure 3:**
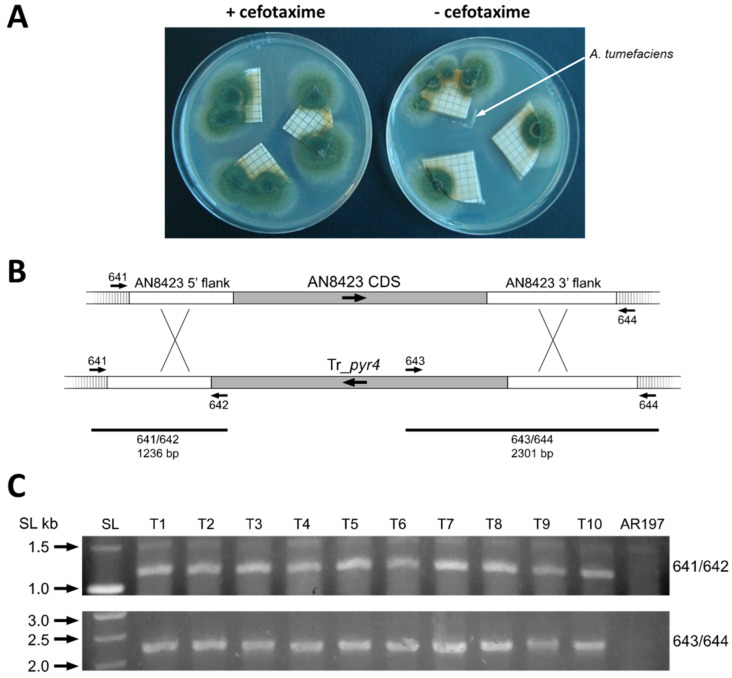
Deletion of AN8423. (**A**) Growth of *A. nidulans* ATM transformants in the presence or absence of cefatoxime. *A. nidulans* conidia at a titre of 10^8^ per mL were used for co-culture. (**B**) Scheme showing replacement of the AN8423 CDS by the Tr_*pyr4* expression cassette. (**C**) Diagnostic PCR with primer pairs 641/642 and 643/644 yields fragments of the sizes predicted for replacement of locus AN8423 by Tr_*pyr4*. SL: Smart Ladder (Promega, Madison, WI, USA).

The antibiotic cefotaxime has been used in other studies to kill *Agrobacterium* during the selection of fungal transformants generated by ATMT [6,7,21,22]. We therefore examined its influence on the transformation of *A. nidulans* (using the optimised condition noted above) by transferring co-culture filters to fungal selection plates (MM + pyridoxine) containing or lacking this antibiotic (Figure 3A). After incubation at 37 °C to promote the growth of *A. nidulans*, no noteworthy difference in the numbers of transformants was seen between its presence or absence. Although more bacterial growth occurred in the absence of cefotaxime, this did not cause problems with the isolation of transformant colonies, indicating that it could be omitted from the selection plates.

Of the uridine prototrophic transformants obtained (see above), ten (T1–T10) were selected for analysis by PCR on material released from lysed conidia. Using two independent sets of oligonucleotide primer pairs (641/642 and 643/644) designed to detect correctly targeted integration events (Figure 3B), the lengths of the amplified fragments in all ten transformants were found to correspond to those predicted for integration of the deletion cassette at the AN8423 locus with consequent replacement of its CDS by Tr_*pyr4* (Figure 3C); these fragments were absent in the non-transformed control AR197.

The above demonstrates deletion of a gene by ATMT in conidia of an *A. nidulans* NHEJ mutant, achieved through complementation of the *pyrG89* mutation by the Tr_*pyr4* cassette cloned in a modified binary vector. The use of *A. nidulans* conidia (uninucleate) obviates the need to perform serial rounds of purification of protoplast-derived transformants.

### 3.2. A Visual Reporter of Transformation

Complementation of a deletion mutant phenotype by an ectopically integrated functional (wild-type) allele is used to confirm a direct relationship between the deleted gene and the phenotype observed and thus exclude the influence of fortuitous mutations [14,23,24]. Since position effects are known to influence gene expression [25], it could be beneficial for comparative analyses to establish a specific genomic locus as a target for ectopic integrations. The *wA* locus (AN8209) in *A. nidulans* encodes a polyketide synthase involved in the biochemical pathway of conidial pigmentation [26,27,28]. Loss of function of this gene impedes pigment development resulting in white conidia, and no other prejudicial metabolic consequences have been reported. In combination with a suitable viability-related marker for the selection of transformants, the phenotype associated with disruption of this locus (white conidia) would also enable visual confirmation of site-specific integration events, and this would be particularly useful for transformation of *nkuA*^+^ strains.

**Figure 4:**
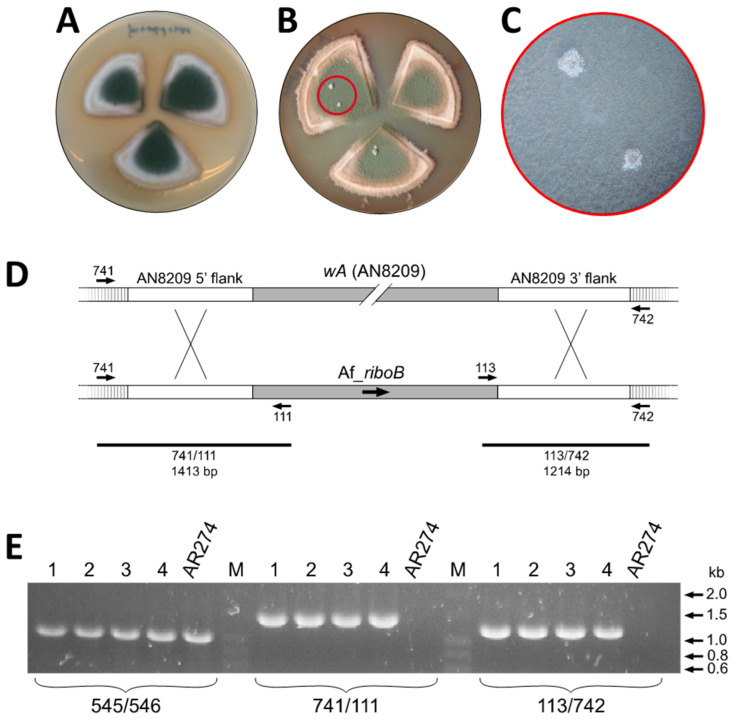
Integration of Af_*riboB* in the *wA* locus. (**A**) Growth of AR198 on MM plates supplemented with pyridoxine but lacking riboflavin. (**B**) Late appearance of white colonies on MM plates lacking riboflavin. (**C**) Approximately 20× magnification of the red-circled area in (**B**). (**D**) Scheme showing insertion of the Af_*riboB* expression cassette in *wA*. (**E**) Diagnostic PCR with primer pairs 741/111 and 113/742 yielded fragments of the sizes predicted for insertion of Af_*riboB* in the *wA* locus. Numbers refer to the four independent transformants analysed.

For the purpose of directing transforming DNA sequences to the *wA* locus, a construct was generated comprising the *A. fumigatus riboB* expression cassette (Af_*riboB*; [13]) flanked by the upstream and downstream sequences (~1 kb of each) of the *A. nidulans wA* gene (i.e., 5′AN8209-Af_*riboB*-3′AN8209); this was then cloned into the unique *Sma*I site of the *kan^R^* binary vector pCAMBIA-mini^AseI^ (derived from pCAMBIA1300 by complete digestion with *Ase*I to eliminate the *hph* gene, the entire CaMV 35S promoter and the CaMV polyA signal; Figure 1A), yielding plasmid pCmAdelwA (Figure 1C; details in Materials and Methods) that was then used to transform *A. tumefaciens* strain AGL1 by electroporation. Transformed bacterial cells selected on the basis of their acquisition of kanamycin resistance were then co-cultivated with conidia of *A. nidulans* NHEJ mutant strain AR198 on nitrocellulose filters under the conditions previously determined for ATMT. The filters were subsequently cut into quadrants, placed on fungal MM selection plates (supplemented with pyridoxine and cefotaxime but lacking riboflavin), and incubated at 37 °C. Over the following days the filters were examined by simple visual inspection and low-magnification microscopy (~20 fold). Whereas the appearance of discrete colonies had been observed in the previous transformations using Tr_*pyr4* as the selectable marker, mycelial growth on this occasion was seen to extend over the entirety of the filters as time progressed, ultimately yielding a uniform lawn of green-conidiating fungal biomass (Figure 4A).

Given this unexpected result, conidia were carefully picked from the filter quadrants by gently touching the surface of the conidial lawn with a loop and were streaked onto pyridoxine-supplemented MM agar. Like the conidia of AR198, these failed to germinate and were therefore auxotrophic for riboflavin. Thus, the mycelial growth on the filter quadrants occurred in the absence of the desired transformation (no white conidia) and was not due to heritable complementation of the riboflavin auxotrophy.

The MM selection plates carrying the co-culture filters were maintained at room temperature and periodically inspected. Four to five weeks after the transformation a few white pinpoints in the green conidial lawn became discernible by eye (Figure 4B). Low-magnification microscopy of these confirmed the presence of a few conidiophores bearing only white conidia (Figure 4C) and also revealed the emergence of other white colonies, including some at filter edges, the hyphae of which extended beyond the filter and into the surrounding medium. With the aid of the microscope, four white regions were touched lightly with a loop to recover white conidia that were subsequently streaked onto plates of MMA supplemented with pyridoxine. Mycelia from these inocula grew and only developed white conidiophores, a result consistent with inherited riboflavin prototrophy (complementation) and disruption of the *wA* locus.

The structure of the *wA* locus in several white colonies of independent origin was analysed by PCR. gDNA isolated from clonally pure liquid-grown mycelia was used as template in conjunction with primer pairs 111/741 and 113/742 that were designed to yield products diagnostic of specific integration of the transforming DNA (5′AN8209-Af_*riboB*-3′AN8209) in *wA* (Figure 4D). In all four cases the PCR products of both primer pairs were consistent with integration of Af_*riboB* in *wA,* whereas no products were seen for gDNA prepared from the wild-type strain AR274; a control PCR performed with primers 545/546 corresponding to an unrelated locus (AN9425/*lraD*; [24]) yielded the expected DNA product (1024 bp) in AR274 as well as in all four ATMT transformants (see Figure 4E).

### 3.3. Nutrient Cross-Feeding Nullifies Certain Selection Systems

The ATMT experiments reported above using two nutritionally selectable genes manifested surprisingly different results. Whereas discrete Tr_*pyr4* transformant colonies were apparent just a few days after the transfer of co-culture filters to fungal selection plates, Af_*riboB* transformants were detected weeks later, independently of the nutritional selection marker, on filters overgrown by the non-transformed parental strain. Thus, unlike the auxotrophy for uridine, riboflavin auxotrophy was masked in the presence of *A. tumefaciens,* indicating that the bacterium may act as a source of this vitamin and cross-feed the fungus during co-culture.

**Figure 5:**
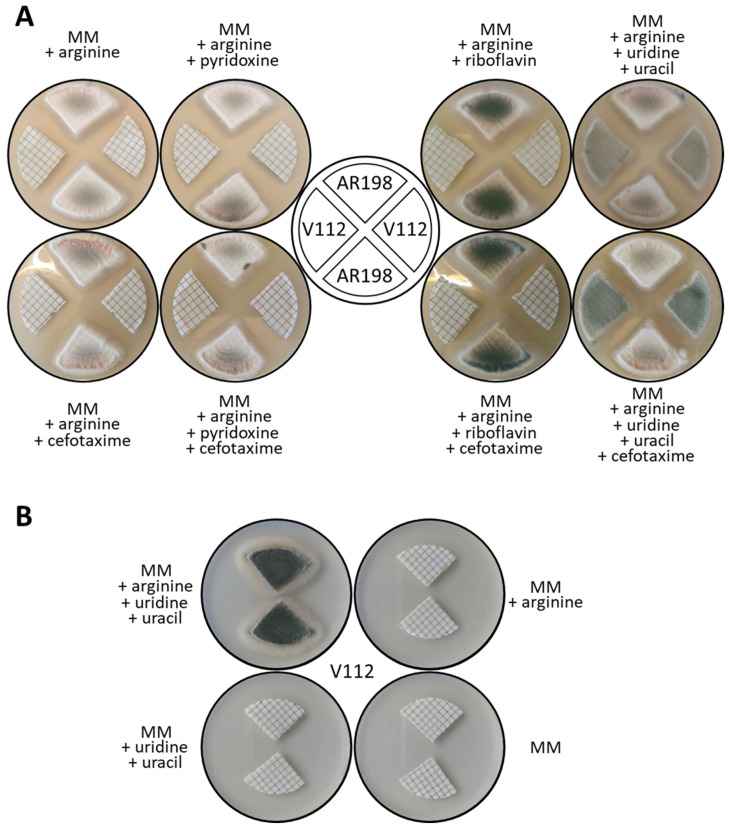
Nutrient cross-feeding. (**A**) Suppression of riboflavin and pyridoxine auxotrophies by *A. tumefaciens*. Relevant genotypes: AR198 (*riboB2*, *pyroA4*) and V112 (*pyrG89*, *argB2*). (**B**) Arginine auxotrophy in V112 is not suppressed by *A. tumefaciens*.

To further assess the cross-feeding hypothesis, co-cultures of *A. tumefaciens* AGL1 (i.e., not carrying a binary vector) with conidia of two *A. nidulans* strains (AR198 and V112) carrying different auxotrophies were set up as ‘mock’ transformations on nitrocellulose filters. After incubation on *At*-IM plates under co-culture conditions the filters were cut and transferred to MM fungal selection plates supplemented with appropriate nutrients and examined after incubation at 37 °C for 2 days. Whilst the growth of AR198, auxotrophic for both riboflavin (*riboB2*) and pyridoxine (*pyroA4*), on co-culture filters placed on pyridoxine-supplemented MM plates confirmed the suppression of riboflavin auxotrophy observed previously, its ability to grow on filters placed on MM plates supplemented with riboflavin also revealed suppression of pyridoxine auxotrophy in the presence of the bacterium (Figure 5A). By contrast the *pyrG89*, *argB2* mutant (V112) failed to grow on MM despite the presence of bacteria unless the medium was supplemented with uridine/uracil, thus confirming the inability of *A. tumefaciens* to suppress uridine/uracil auxotrophy. Cefatoxime plays no role in the observed suppression, as no differences in growth of either fungal strain were observed between plates containing or lacking the antibiotic.

The potential utility of arginine auxotrophy (due to the *argB2* mutation) in ATMT was also tested in co-cultures of V112 with AGL1 (Figure 5B). As expected, no growth of V112 was observed on MM, whereas normal growth occurred on fully supplemented (i.e., uridine/uracil + arginine) medium. Supplementation with uridine/uracil alone failed to result in growth, demonstrating that *A. tumefaciens* does not serve as a source of arginine for the fungus. Like *pyrG89*, the *argB2* allele thus has the potential for use as a selectable marker in ATMT.

## 4. Discussion

Although the transformation of protoplasts remains widely used for the introduction of genetic material into *A. nidulans*, it is nevertheless a technique that has a number of pitfalls. The ease or difficulty with which transformable protoplasts can be obtained is influenced by several factors including natural variations between *A. nidulans* strains, the type of biomass to be used and its growth conditions, variations between batches of protoplasting enzymes that affect both protoplast release and viability, as well as other considerations related to the osmotic fragility of the latter. Osmotic stabilization is often achieved with buffers having high sugar or sugar alcohol concentrations, and their influence on carbon catabolite repression (CCR) could lead to effects on gene expression that could impact the selection of transformants. ATMT is an alternative method of transformation that has found wide use in pathogenic fungi and has been applied to different fungal structures including conidia. In the current study we have demonstrated the potential of NHEJ mutant conidia carrying certain auxotrophic markers for achieving efficient site-specific integration in the *A. nidulans* genome by ATMT and selection of the resulting transformants based on nutrient utilisation, thus avoiding the difficulties noted above associated with the transformation of protoplasts. Whilst impossible to compare directly due to the very different natures of the experimental methodologies, under our conditions the number of prototrophic ATMT transformants obtained per experiment is comparable to that for transformants obtained in a ‘typical’ transformation of Δ*nkuA* protoplasts. ATMT of conidia avoids the difficulties associated with protoplast transformation and facilitates the direct isolation of stable primary transformants by virtue of the uninuclear nature of conidia. The use of conidia does, however, have a limitation in that they are not amenable to the analysis of essential genes since, unlike the situation with protoplasts, no heterokaryotic stage occurs in which nuclei deleted for essential genes can be maintained for phenotypic characterisation by the presence of untransformed nuclei (heterokaryon rescue, [29]).

Decades of genetic research in *A. nidulans* have resulted in a rich legacy of auxotrophic mutants and the identification of many of the genes responsible, and in a number of cases ‘nutritional’ expression systems have been developed as excellent selectable markers for genetic transformation. Interestingly, our study has highlighted an important restriction regarding the application of certain nutritional markers in ATMT. Whilst the auxotrophies for riboflavin and pyridoxine (conferred by the *riboB2* and *pyroA4* mutant alleles, respectively) are successful as selection systems in other transformation techniques —and are included in various NHEJ strains [13]—we have found them to be unusable for counter-selection of the fungal host in ATMT. Our observations have shown that the fungus is able to cross-feed both of the associated nutritional requirements from the bacterial cells present during co-culture, resulting in suppression of these auxotrophies and growth of the non-transformed strain. By contrast, this was not the case for uridine/uracil auxotrophy conferred by the *pyrG89* allele. The lack of growth of the strain carrying this auxotrophy revealed the absence or insufficiency of uridine/uracil cross-feeding, thus permitting direct isolation of the corresponding prototrophic transformants. The explanation for the different behaviours of the auxotrophies in ATMT may reside in the scale of the demand for the respective nutrients. Riboflavin and pyridoxine are vitamins, and hence required in small quantities, whereas orotidine-5′-phosphate decarboxylase (encoded by *pyrG* and Tr_*pyr4*) is necessary for the synthesis of pyrimidine nucleotides that will be required in considerable quantity for building nucleic acids during fungal growth. Similarly, the scale of the arginine requirement for protein synthesis in the growing fungus could be the explanation for the apparent absence of cross-feeding of the arginine auxotroph (*argB2*) in the presence of the bacterium. Thus, strains carrying the *argB2* mutation could also be used for obtaining *A. nidulans* transformants by ATMT.

We have used ATMT on the one hand to delete a genomic locus (AN8423 was our chosen candidate) and on the other to introduce a recombinant DNA fragment at a specific genomic site (*wA*) that results in an easily distinguishable visible phenotype and obviates the need for diagnostic PCR to confirm transformant identity. In both cases integration of the selectable cassette (Tr_*pyr4* and Af_*riboB*) in the NHEJ strains was achieved at high efficiency since all the transformants analysed by PCR were shown to have the desired gene deletion/disruption. However, an interesting consequence of the failure to select riboflavin prototrophic transformants (due to cross-feeding of this vitamin) carrying the Af_*riboB* cassette directed to the *wA* locus was their eventual detection by visual means, specifically the development of white conidia, thus demonstrating the identification of transformants without recourse to a nutritional marker. Isolation of the transformants benefitted from the uninuclear nature of *A. nidulans* conidia and was easily effected by careful use of an inoculation loop under low-power microscopy. Although the auxotrophic selection marker used (*riboB2*) was rendered unusable in the presence of bacterial cells, that ceased to be the case upon plating of white conidia in the absence of the bacterium. Harvesting conidia, after the formation of the conidial lawn, and their subsequent wash and inoculation by spreading at low density onto MM fungal selection plates containing cefotaxime may provide a means to enrich and facilitate the visual identification and isolation of white conidial transformants. Thus, integration at the *wA* locus could also be useful in strains that lack an auxotrophic marker or in cases where such a marker might be undesirable.

The last few years have seen the increasing application of CRISPR/Cas technology in filamentous fungi, and whilst protoplast transformation has been the most commonly used method to introduce the Cas and guide RNA modules into the fungal cell, a few instances of the use of ATMT (alone or in conjunction with protoplast transformation) have also been reported [30,31]. The elements of ATMT in *A. nidulans* that we present here could have potential as a platform for the development and application of CRISPR/Cas-based tools for modifying the structure of the *A. nidulans* genome (Cas9/Cas12a) and/or modulating the expression of individual genes or gene sets (CRISPRa/CRISPRi), preferably in a single transformation event.

## Figures and Tables

**Table 1:** *Aspergillus nidulans* strains used in this study.

Strain Code	Name	Genotype ^1^	References
AR197	TN02A3	Δ*nkuA::argB*, *argB2*, *pyrG89*, *pyroA4*	FGSC A1149
AR198	TN02A21	Δ*nkuA::argB*, *argB2*, *riboB2*, *pyroA4*	[13]
AR271		Δ*nkuA*::*argB, argB2*, Δ*riboB2*::Af_*riboB, pyroA4*	[14]
AR274	FGSC A4		[15]
V112		*pyrG89*, *argB2*	Lab collection

^1^ All strains carry the mutant allele *veA1* except AR274.

## Data Availability

The data presented in this study are available on request from the corresponding author.

## Supplementary Materials

The following are available online at https://www.mdpi.com/article/10.3390/jof7110961/s1, Table S1: Solutions and media used for ATMT of *A. nidulans* conidia; Table S2: Oligonucleotide primers used.
