# Supplementary material for: Agrobacterium tumefaciens-Mediated Transformation of NHEJ Mutant Aspergillus nidulans Conidia: An Efficient Tool for Targeted Gene Recombination Using Selectable Nutritional Markers
Source: J Fungi (Basel). 2021 Nov 12;7(11):961. doi: 10.3390/jof7110961 (PMC8623315; doi:10.3390/jof7110961)
Supplement: Supplementary file 1 [file jof-07-00961-s001.zip › Supplementary Table S1.pdf]

Table S1. Solutions and media used for ATMT of *A. nidulans* conidia.

**Solution**	***At*-minimal medium (*At*-MM) 100 ml**	***At*-induction medium (*At*-IM) 100 ml**	**Solid *At*-IM for co-culture 1 litre**
Buffer K	1 ml	1 ml	10 ml
Buffer M-N	2 ml	2 ml	20 ml
$CaCl_2$ 1 %	100 µl	100 µl	1 ml
Glucose (20%)	1 ml	500 µl	5 ml
NH_4NO_3 (20%)	250 µl	250 µl	2.5 ml
MES (1M)	-	4 ml	40 ml
Glycerol (50%)	-	1 ml	10 ml
Bacteriological agar	-	-	15 g
Sterile distilled water	94.05 ml	89.45 ml	894.5 ml *
$FeSO_4$ (0.01%)	1 ml	1 ml	10 ml
Trace elements	500 µl	500 µl	5 ml
Acetosyringone (200 mM)	-	100 µl	1 ml
Kanamycin (50 mg/ml)	100 µl	100 µl	1 ml

* Liquid *At*-MM and *At*-IM were prepared by adding the sterilized components to sterile distilled water under aseptic conditions; for solid *At*-IM, the agar was dissolved and autoclaved in distilled water and the sterilized components were added during cooling (~50ºC) prior to pouring plates.

Buffer K (100 ml): 20 g K_2HPO_4; 14.5 g KH_2PO_4; pH 7. Sterilize by autoclaving; store at 4°C.

Buffer M-N (100 ml): 3 g $MgSO_4.7H_2O$; 1.5 g NaCl. Sterilize by autoclaving; store at 4°C.

1% $CaCl_2$: Prepare from a concentrated stock in sterile distilled water. Store at 4°C. Sterilize concentrated stock by filtration and store at room temperature.

20% NH_4NO_3 (100 ml): 20 g NH_4NO_3. Sterilize by autoclaving; store at 4°C.

20% Glucose (100 ml): 20 g glucose. Sterilize by autoclaving; store at 4°C.

$FeSO_4.7H_2O$ (100 ml): 0.01 g $FeSO_4.7H_2O$. Prepare from a concentrated stock in distilled water. Sterilize the stock with two to three 10 second pulses in the microwave and store at 4°C in darkness.

1M MES (2-(N-Morpholino) ethanesulfonic acid) (100 ml): 19.52 g; pH 5.3. Sterilize by filtration, aliquot and store at -20°C. (Sigma Ref. M8250-25G).

50% (v/v) Glycerol (100 ml): 50 ml of 100% glycerol. Sterilize by autoclaving; store at 4°C.

Trace element solution (1000 ml): 100 mg $ZnSO_4.7H_2O$; 100 mg $CuSO_4.5H_2O$; 100 mg H_3BO_3; 100 mg $MnSO_4.H_2O$; 100 mg $Na_2MoO_4.2H_2O$. Sterilize by filtration; store at 4°C.
