# Supplementary material for: Agrobacterium tumefaciens-Mediated Transformation of NHEJ Mutant Aspergillus nidulans Conidia: An Efficient Tool for Targeted Gene Recombination Using Selectable Nutritional Markers
Source: J Fungi (Basel). 2021 Nov 12;7(11):961. doi: 10.3390/jof7110961 (PMC8623315; doi:10.3390/jof7110961)
Supplement: Supplementary file 1 [file jof-07-00961-s001.zip › Supplementary Table S2.pdf]

Table S2. Oligonucleotide primers used.

**Number**	**Primer name** - restriction site(s)	**Primer sequence***
111	Afrib-1T	5'-GTGCGATTACATGAAGGTCC-3'
113	Afrib-2T	5'-TGCTCGTCACACTCATGTAACG-3'
545	AN9425-3' F	5’-AGCCTCGCGACGGATATTAAG-3’
546	AN9425-3' Rev - *Hae*II	5’-ACTATGGCGCTGTTGGCAGAC-3’
551	AN8209-wA 5' F - *Bam*HI	5’-ggttaggatccGTGTGATCATCTGAGCTCTGG-3’
552	AN8209-wA 5' Rev - *Eco*RI	5’-aagggaattcgatATCAGGAGAAGGAGAGTCAAGTCC-3’
553	AN8209-wA 3' F - *Eco*RI	5’-aagggaattcTTGTGAGCGCCGGTTGGTCTG-3’
554	AN8209-wA 3' Rev - *Sal*I, *Not*I	5’-cacaagtcgacgcggccgCAATGCTGCTGCGCACTCTGG-3’
573	AN8423-5' F - *Kpn*I, *Spe*I	5'-gaaggtaccactagtCAGCCGAACTCTCAAACTTTC-3'
574	AN8423-5' Rev - *Sal*I	5'-acggaggtcgacGTGCCCTTAATTGTGCTACTG-3'
575	AN8423-3' F - *Sal*I	5'-acggaggtcgacGTGTTGCCTACTCCTGAAATG-3'
576	AN8423-3' Rev - *Spe*I	5'-tacaggactagtGCATCATTGGTCTAGTGGTAG-3'
641	AN8423 5' outer	5'-ATTCAGTGTTTGGCAGCCGTGG-3'
642	Tr_pyr4 3' out	5'-ATTTGCTTTTGCGCGTGGAGG-3'
643	Tr_pyr4 5' out	5'-TCTGCTGTGCGAATGACGTTGG-3'
644	AN8423 3' retuo	5'-TGACTGCGGGATTGAGTGATGG-3'
741	wA 5 outer	5'-TGAAGCTACAGAGTTACGACC-3'
742	wA 3 retuo	5'-ACAACAGAGAATGCAGCACTGG-3'

* Restriction enzyme recognition sites are specified and underlined; capital letters correspond to gene-specific sequences.
